# Prognostic value and preoperative predictors of microvascular invasion in solitary hepatocellular carcinoma ≤ 5 cm without macrovascular invasion

**DOI:** 10.18632/oncotarget.18049

**Published:** 2017-05-22

**Authors:** Hui Zhao, Ye Hua, Zhihua Lu, Shen Gu, Laifa Zhu, Yuan Ji, Yudong Qiu, Tu Dai, Huihan Jin

**Affiliations:** ^1^ Department of Hepatopancreatobiliary Surgery, Nanjing Medical University Affiliated Wuxi Second People’s Hospital, Wuxi, Jiangsu, China; ^2^ Department of Neurology, Nanjing Medical University Affiliated Wuxi Second People’s Hospital, Wuxi, Jiangsu, China; ^3^ Department of Hepatopancreatobiliary Surgery, The Affiliated Drum Tower Hospital of Nanjing University Medical School, Nanjing, Jiangsu, China

**Keywords:** hepatocellular carcinoma, microvascular invasion, prognosis, preoperative predictors

## Abstract

**Objectives:**

The aim of this study was to investigate the prognostic value and preoperative predictors of microvascular invasion (MVI) in solitary hepatocellular carcinoma (HCC) ≤ 5 cm without macrovascular invasion.

**Methods:**

A total of 233 consecutive HCC patients underwent curative hepatectomy were included in our study. Independent risk factors influencing the prognosis were identified, and preoperative predictors for MVI were determined.

**Results:**

Multivariate regression analysis identified ICG-R15, BCLC staging and MVI as independent risk factors for the overall survival rate. Type of resection and MVI were independent risk factors for the recurrence-free survival rate. Kaplan-Meier analysis showed the overall survival and recurrence-free survival rates in patients with MVI were significantly poorer than those in patients without MVI (*P* = 0.002 and *P* = 0.001). Anatomical resection obviously improved the overall survival and recurrence-free survival rates in patients with MVI compared with non-anatomical resection (*P* = 0.017 and *P* = 0.009). A prediction scoring system for MVI was built up according to the three independent predictors (tumor size > 3.5 cm, AFP > 200 ng/mL and GGT > 53 U/L). The prevalence of MVI in HCC patients with predictive score ≥ 2 was 58.3%, which was obviously higher than patients with predictive score < 2 (20.8%).

**Conclusions:**

MVI is associated with a poor prognosis in solitary HCC ≤ 5 cm after hepatectomy. Anatomical resection could improve the prognosis of HCC patients with MVI. The preoperative prediction scoring model has practical value for the prediction of MVI.

## INTRODUCTION

Hepatocellular carcinoma (HCC) is the sixth most common malignancy and the third leading cause of cancer-related death worldwide [1]. Due to recent advances in diagnosis and surgical technology, curative hepatectomy is now widely considered as the first choice of therapy for HCC with well liver functional reserve, especially early-stage HCC [2]. However, even with these advances, the long-term outcomes of HCC remain unsatisfactory due to the high postoperative recurrence [3]. Approximately 70% of HCC patients have a recurrence within the 5 years after curative hepatectomy [4].

Vascular invasion is generally considered as an important risk factor for the prognosis of HCC patients after curative hepatectomy [5]. Macrovascular invasion, which could be evaluated by macroscopic examination or preoperative imaging, is helpful to provide a basis for selecting rational therapy. However, microvascular invasion (MVI) is difficult to be used for evaluating the prognosis of HCC preoperatively because it is only confirmed after operation by histopathological diagnosis [6]. MVI, defined as the invasion of tumor cells in intrahepatic portal vein or hepatic vein branches, is the beginning of intrahepatic dissemination and metastasis in HCC [5]. The prevalence of MVI ranges from 15% to 57% in HCC specimens [7]. Although the formation mechanism of MVI is not clear, previous researches have identified MVI is associated with poor prognosis of HCC patients after hepatectomy [8, 9]. To our knowledge, MVI is more common in advanced HCC patients presented with large size (tumor size > 5 cm) and multiple lesions [10]. But there are few researches focused on the influence of MVI on the prognosis of early-stage HCC (solitary, tumor size ≤ 5 cm, without macrovascular invasion). In the present research, we aimed to investigate the prognostic outcomes and preoperative predictors of MVI in early-stage HCC patients after curative hepatectomy.

## RESULTS

### Clinicopathological characteristics and long-term survival

The baseline characteristics of all HCC patients were presented in Table 1. Overall, the median age was 55 years (range 22-87 years). Of 233 HCC patients, 185 patients (79.3%) were male and 48 patients (20.7%) were female respectively. Positive HBsAg and liver cirrhosis were presented in 183 patients (78.5%) and 159 patients (68.2%). The median preoperative ICG-R15 was 5.0% (range 0.5-31.5%). A total of 119 patients (51.1%) received anatomical liver resection and 114 patients (48.9%) received non-anatomical liver resection. The median tumor size was 3.5 cm (range 1.0-5.0 cm). Forty-five patients (19.3%) and 164 patients (70.4%) were diagnosed with well and moderate differentiated HCC, respectively. MVI was found in 87 patients (37.3%). The median follow-up time was 49 months (range 2-142 months). No deaths occurred in hospital. The 1-, 3-, and 5-year overall survival rates for the entire cohort were 91.4%, 73.7%, and 63.5%, respectively. The 1-, 3-, and 5-year recurrence-free survival rates for the entire cohort were 78.0%, 55.8%, and 39.2%, respectively.

**Table 1:** Clinicopathological characteristics of the overall cohort

Variable	Overall cohort (n = 233)
Age (years) ^a^	55 ± 12
Gender	
Male	185 (79.3)
Female	48 (20.7)
HBsAg	
Positive	183 (78.5)
Negative	50 (21.5)
Background liver	
Noncirrhosis	74 (31.8)
Cirrhosis	159 (68.2)
Child–Pugh grade	
A	226 (97.0)
B	7 (3.0)
BCLC staging	
0	27 (11.6)
A	206 (88.4)
ICG-R15 ^b^	5.0 (0.5-31.5)
ALT (U/L) ^b^	35.5 (7.5-617.1)
AST (U/L) ^b^	32.8 (14.5-285.5)
TB (umol/L) ^b^	15.9 (3.6-47.7)
DB (umol/L) ^b^	4.6 (1.3-27.6)
GGT (U/L) ^b^	40.0 (15.3-683.5)
AKP (U/L) ^b^	77.5 (32.8-534.6)
Albumin (g/L) ^a^	42.2 ± 4.1
INR ^b^	1.1 (0.9-1.8)
Platelets (109/L) ^a^	135 ± 53
AFP (ng/mL) ^b^	66.8 (0.7-62593.0)
Types of resection	
Anatomical	119 (51.1)
Non-anatomical	114 (48.9)
Operation time (min) ^b^	210 (75-410)
Blood loss (mL) ^b^	400 (50-1500)
Transfusion	
Yes	42 (18.0)
No	191 (82.0)
Tumor size (cm) ^b^	3.5 (1.0-5.0)
Tumor differentiation	
Well	45 (19.3)
Moderate	164 (70.4)
Poor	24 (10.3)
Microvascular invasion	
Yes	87 (37.3)
No	146 (62.7)

Parenthesis indicates percentage unless indicated.

^a^ median (range); ^b^ mean ± standard deviation. AFP, alpha-fetoprotein; AKP, alkaline phosphatase; ALT, alanine aminotransferase; AST, aspartate aminotransferase; TB, total bilirubin; DB, direct bilirubin; GGT, gamma glutamyl transpeptidase; INR, international normalized ratio.

### Univariate and multivariate analysis of prognostic risk factors for the entire cohort

In univariate analysis, Child–Pugh grade, ICG-R15, BCLC staging, tumor size, type of resection and MVI significantly influenced the overall survival rate (Table 2). Additionally, the level of GGT, blood loss, type of resection and MVI significantly influenced the recurrence-free survival rate (Table 3). Cox multivariate regression analysis identified ICG-R15 (HR = 1.081, 95% CI 1.033-1.132, *P* = 0.001), BCLC staging (HR = 10.244, 95% CI 1.414-74.248, *P* = 0.021) and MVI (HR = 1.783, 95% CI 1.061-2.997, *P* = 0.029) as independent risk factors for the overall survival rate. While type of resection (HR = 1.444, 95% CI 1.049-1.988, *P* = 0.024) and MVI (HR = 1.670, 95% CI 1.212-2.302, *P* = 0.002) were independent risk factors for the recurrence-free survival rate.

**Table 2:** Univariate and multivariate analysis of risk factors for overall survival rate

Variable	Univariate analysis		Multivariate analysis	
	HR (95%CI)	*P*-value	HR (95%CI)	*P*-value
Age	1.010 (0.991-1.029)	0.303		
Gender (male vs. female)	1.111 (0.637-1.938)	0.712		
HBsAg (negative vs. positive)	0.917 (0.561-1.499)	0.729		
Background liver (noncirrhosis vs. cirrhosis)	0.938 (0.585-1.504)	0.789		
Child–Pugh (B vs. A)	3.087 (1.248-7.637)	0.015		
ICG-R15	1.084 (1.035-1.135)	0.001	1.081 (1.033-1.132)	0.001
BCLC (A vs. 0)	4.572 (1.444-14.473)	0.010	10.244 (1.414-74.248)	0.021
ALT	0.999 (0.995-1.004)	0.772		
AST	1.000 (0.996-1.005)	0.824		
TB	1.013 (0.994-1.033)	0.186		
DB	1.034 (0.975-1.096)	0.264		
AKP	1.003 (0.999-1.006)	0.154		
GGT	1.002 (1.000-1.004)	0.084		
Albumin	0.974 (0.925-1.026)	0.320		
INR	4.299 (0.764-24.191)	0.098		
Platelet	0.998 (0.994-1.001)	0.246		
AFP	1.000 (1.000-1.000)	0.342		
Tumor size	1.230 (1.073-1.411)	0.003		
Operation time	1.001 (0.999-1.004)	0.253		
Blood loss	1.000 (1.000-1.001)	0.068		
Transfusion (yes vs. no)	0.808 (0.632-1.034)	0.090		
Types of resection (non-anatomical vs. anatomical)	1.619(1.056-2.481)	0.027		
Tumor differentiation (moderate/poor vs. well)	0.815 (0.617-1.077)	0.150		
MVI (yes vs. no)	1.938 (1.271-2.954)	0.002	1.783 (1.061-2.997)	0.029

AFP, alpha-fetoprotein; AKP, alkaline phosphatase; ALT, alanine aminotransferase; AST, aspartate aminotransferase; TB, total bilirubin; DB, direct bilirubin; GGT, gamma glutamyl transpeptidase; INR, international normalized ratio.

**Table 3:** Univariate and multivariate analysis of risk factors for recurrence-free survival rate

Variable	Univariate analysis		Multivariate analysis	
	HR (95%CI)	*P*-value	HR (95%CI)	*P*-value
Age	1.009 (0.995-1.024)	0.218		
Gender (male vs. female)	0.892 (0.604-1.318)	0.566		
HBsAg (negative vs. positive)	1.119 (0.754-1.662)	0.576		
Background liver (noncirrhosis vs. cirrhosis)	1.038 (0.732-1.473)	0.833		
Child–Pugh (B vs. A)	0.582 (0.257-1.319)	0.195		
ICG-R15	1.125 (0.974-1.182)	0.216		
BCLC (A vs. 0)	1.473 (0.850-2.553)	0.168		
ALT	1.001 (0.999-1.003)	0.193		
AST	1.005 (1.003-1.008)	0.034		
Total bilirubin	1.003 (0.987-1.020)	0.693		
Direct bilirubin	1.016 (0.969-1.064)	0.514		
AKP	1.002 (0.999-1.004)	0.241		
GGT	1.004 (1.001-1.006)	0.002		
Albumin	0.968 (0.930-1.006)	0.101		
INR	1.000 (0.208-4.800)	0.998		
Platelet	1.000 (0.997-1.003)	0.986		
AFP	1.000 (1.000-1.000)	0.447		
Tumor size	1.095 (0.987-1.214)	0.087		
Operation time	1.001 (1.000-1.003)	0.116		
Blood loss	1.000 (1.000-1.001)	0.032		
Transfusion (yes vs. no)	0.918 (0.755-1.117)	0.393		
Types of resection (NAR vs. AR)	1.408 (1.023-1.937)	0.006	1.444 (1.049-1.988)	0.024
Tumor differentiation (moderate/poor vs. well)	0.879 (0.718-1.076)	0.210		
MVI (yes vs. no)	1.661 (1.206-2.228)	0.001	1.670 (1.212-2.302)	0.002

AFP, alpha-fetoprotein; AKP, alkaline phosphatase; ALT, alanine aminotransferase; AST, aspartate aminotransferase; TB, total bilirubin; DB, direct bilirubin; GGT, gamma glutamyl transpeptidase; INR, international normalized ratio.

### Comparisons of long-term survival according to MVI

**Figure 1:**
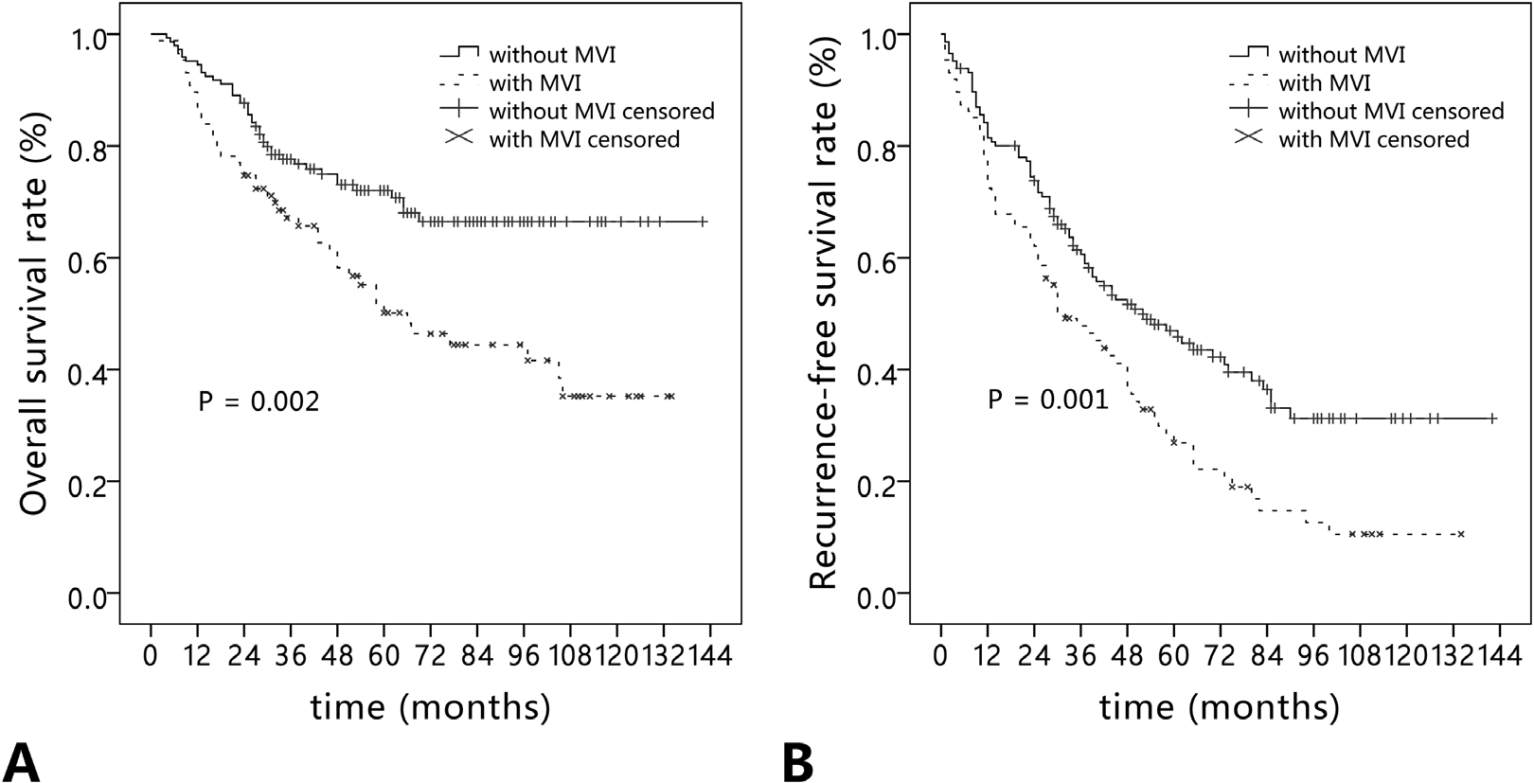
Long-term outcomes in hepatocellular carcinoma patients with (n = 87) and without microvascular invasion (n = 146) **(A)** Overall survival; **(B)** recurrence-free survival.

**Figure 2:**
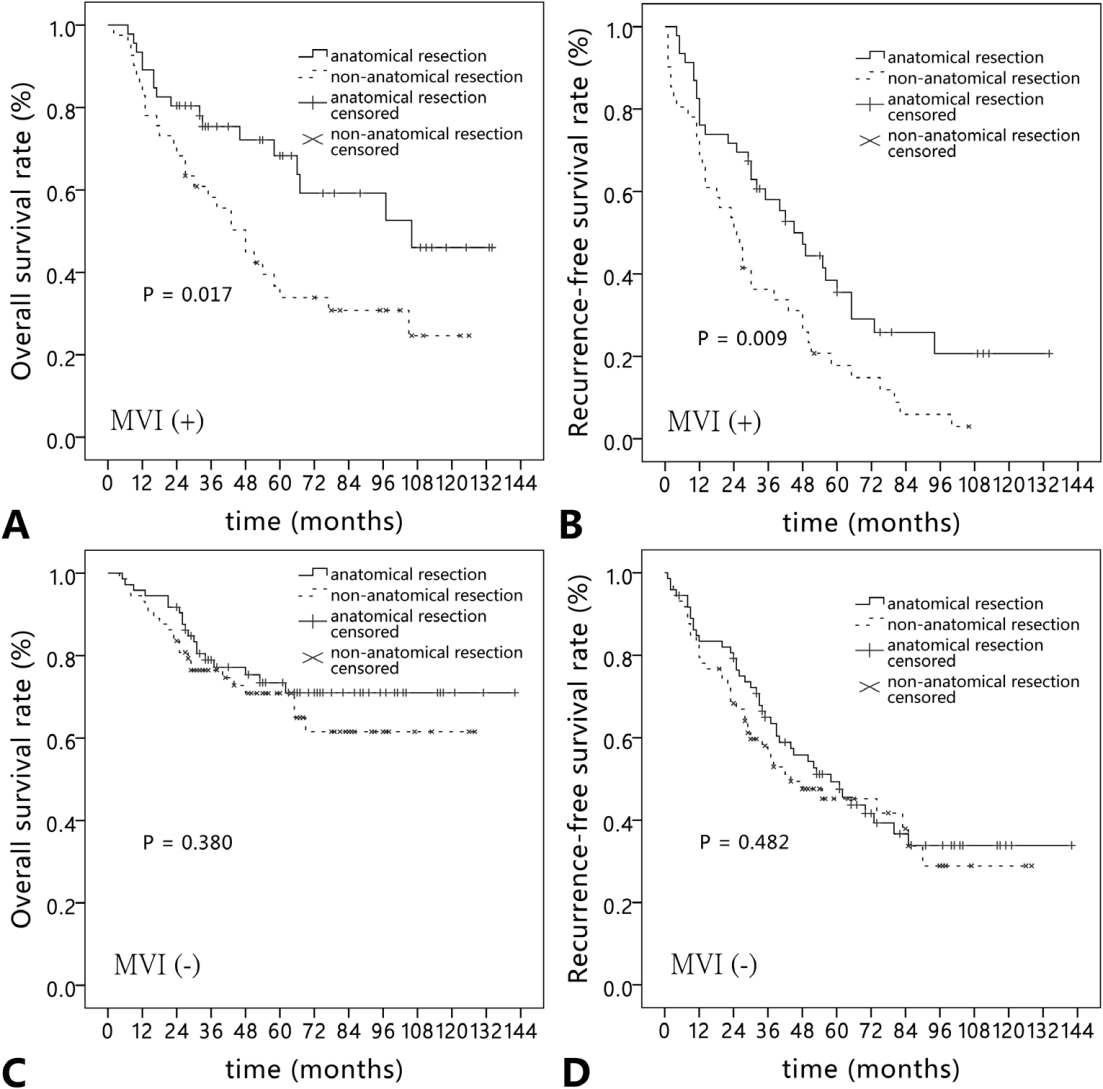
Comparison of **(A, C)** overall survival rate and **(B, D)** recurrence-free survival rate in hepatocellular carcinoma patients with and without microvascular invasion according to types of liver resection (anatomical resection vs. non-anatomical resection).

In HCC patients with MVI (n = 87), the 1-, 3-, and 5-year overall survival rates were 86.2%, 67.1%, and 50.1%, respectively. Correspondingly, the 1-, 3-, and 5-year recurrence-free survival rates were 72.4%, 47.8%, and 26.9%, respectively. In HCC patients without MVI (n = 146), the 1-, 3-, and 5-year overall survival rates were 94.5%, 77.7%, and 72.0%, respectively. Correspondingly, the 1-, 3-, and 5-year recurrence-free survival rates were 81.4%, 60.6%, and 47.0%, respectively. Kaplan-Meier analysis showed the overall survival and recurrence-free survival rates in patients with MVI were significantly poorer than that in patients without MVI (*P* = 0.002 and *P* = 0.001) (Figure 1). In the subgroup analysis according to type of resection, anatomical resection obviously improved the overall survival and recurrence-free survival rates in patients with MVI compared with non-anatomical resection (*P* = 0.017 and *P* = 0.009). No significant difference was observed between the two types of resection in patients without MVI (*P* = 0.380 and *P* = 0.482) (Figure 2).

### The recurrence patterns of HCC patients with and without MVI

Table 4 summarizes the recurrence patterns of HCC patients with and without MVI. The recurrence rate (79.3%) in patients with MVI was higher than that (56.8%) in patients without MVI (*P* < 0.001). Compared with patients without MVI, the median time to recurrence in patients with MVI was remarkably shorter (32.0 vs. 52.0 months, *P* = 0.001). Although there were no significant differences in the intrahepatic recurrence, extrahepatic recurrence and numbers of recurrence between patients with and without MVI, the marginal recurrence was significantly higher in patients with MVI (*P* = 0.017). On the contrary, the recurrence in contralateral hemiliver was significantly lower in patients with MVI (*P* = 0.028).

**Table 4:** The recurrence patterns of hepatocellular carcinoma patients with and without microvascular invasion

	MVI (+) (n=87)	MVI (-) (n=146)	*P*-value
Recurrence	69 (79.3)	83 (56.8)	< 0.001
Time to recurrence (months) median (95% CI)	32.0 (19.9-41.1)	52.0 (35.1-68.9)	0.001
Site of recurrence			
Intrahepatic	64 / 69 (92.8)	79 / 83 (95.2)	0.775
Surgical margin	21 / 69 (30.4)	11 / 83 (13.3)	0.017
Ipsilateral hemiliver	28 / 69 (40.6)	35 / 83 (42.3)	0.974
Contralateral hemiliver	15 / 69 (21.7)	33 / 83 (39.8)	0.028
Extrahepatic	5 / 69 (7.2)	4 / 83 (4.8)	0.775
Number of recurrence			
Solitary	43 / 69 (62.3)	58 / 83 (69.9)	0.418
Multiple	26 / 69 (37.7)	25 / 83 (30.1)	

### Predictive value of preoperative clinical factors for MVI

Preoperative clinical factors were performed to predict MVI (Table 5). The significant predictors (*P* < 0.1) inunivariate analysis were entered into the multivariate logistic regression model to identify the valuable independent predictors for MVI. GGT > 53U/L (OR = 2.360, 95% CI 1.287-4.325, *P* = 0.005), AFP > 200 ng/ml (OR = 2.544, 95% CI 1.399-4.628, *P* = 0.002) and tumor size > 3.5cm (OR = 2.938, 95% CI 1.585-5.447, *P* = 0.001) were independent predictors for MVI.

**Table 5:** Predictors for microvascular invasion of hepatocellular carcinoma on univariate analysis

Variable	MVI (+) (n = 87)	MVI (-) (n = 146)	Univariate analysis	
			OR (95%CI)	*P*-value
Age				
> 60 vs. ≤ 60 years	25 / 62	51 / 95	0.751 (0.422–1.336)	0.330
Gender				
Male vs. female	67 / 20	118 / 28	0.795 (0.416–1.519)	0.487
HBsAg				
Positive vs. negative	72 / 15	111 / 35	1.514 (0.772–2.969)	0.228
Child–Pugh grade				
B vs. A	3 / 84	4 / 142	0.789 (0.172-3.610)	0.760
ALT				
> 50 vs. ≤ 50 U/L	25 / 62	42 / 104	0.998 (0.555-1.795)	0.996
AST				
> 50 vs. ≤ 50 U/L	19 / 68	29 / 117	1.127 (0.588-2.162)	0.718
TB				
> 17.1 vs. ≤ 17.1 μmol/L	41 / 46	50 / 96	1.711 (0.995-2.943)	0.052
DB				
> 7.0 vs. ≤ 7.0 μmol/L	13 / 74	16 / 130	1.427 (0.651-3.131)	0.375
GGT				
> 53 vs. ≤ 53 U/L	44 / 43	42 / 104	2.534 (1.458-4.402)	0.001
Albumin				
> 35 vs. ≤ 35 g/L	83 / 4	138 / 8	1.203 (0.351-4.118)	0.769
INR				
> 1.1 vs. ≤ 1.1	24 / 63	27 / 119	1.679 (0.895-3.149)	0.106
Platelets				
> 100 vs. ≤ 100 ×10^9^/L	59 / 28	113 / 33	0.615 (0.340-1.114)	0.109
AFP				
> 200 vs. ≤ 200 ng/mL	46 / 41	44 / 102	2.601 (1.501-4.507)	0.001
Tumor size				
> 3.5 vs. ≤ 3.5 cm	65 / 22	68 / 78	3.389 (1.893-6.069)	< 0.001

AFP, alpha-fetoprotein; AKP, alkaline phosphatase; ALT, alanine aminotransferase; AST, aspartate aminotransferase; TB, total bilirubin; DB, direct bilirubin; GGT, gamma glutamyl transpeptidase; INR, international normalized ratio.

**Figure 3:**
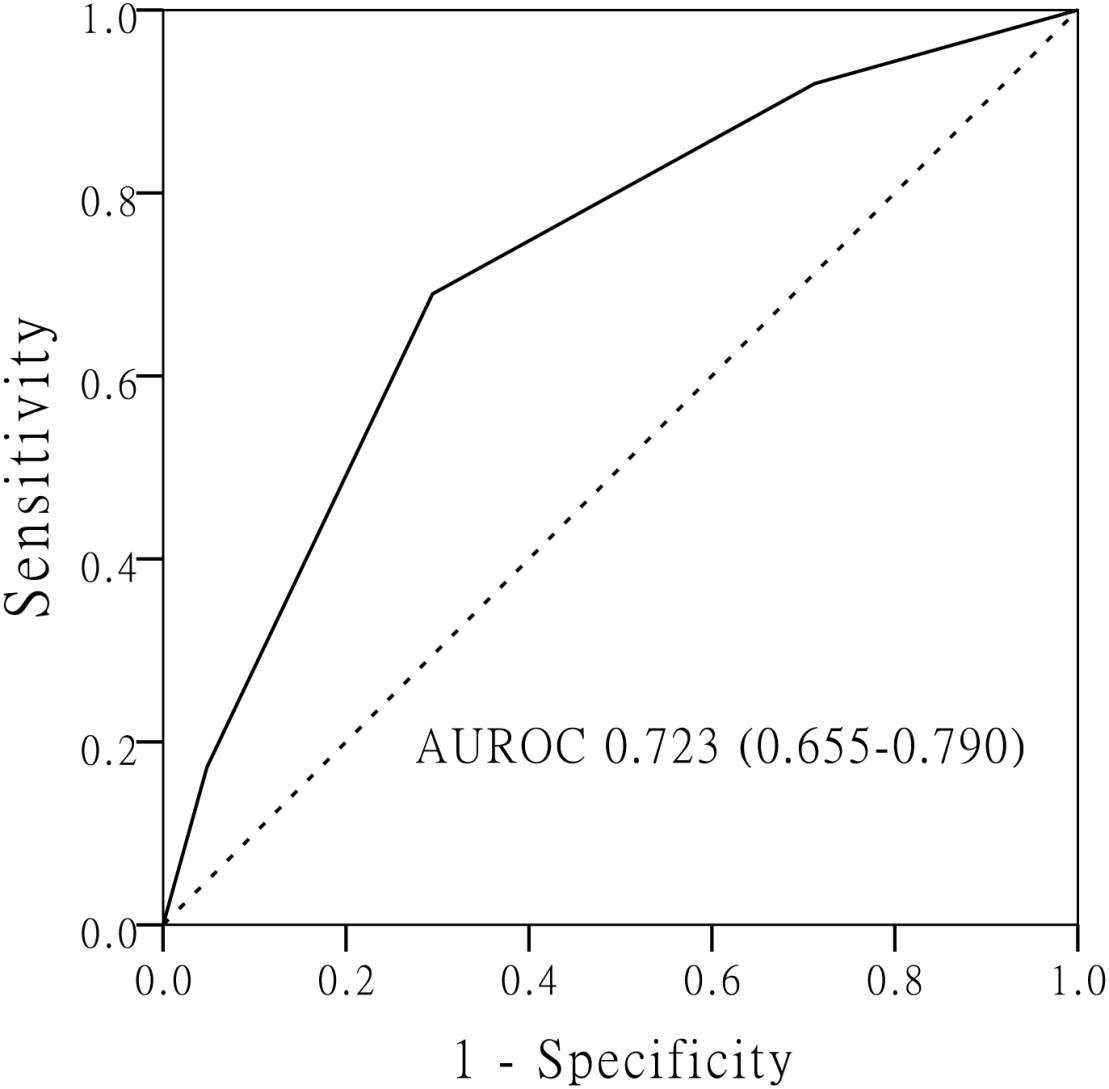
The receiver operating characteristic (ROC) curve of prediction scoring model for microvascular invasion based on the three independent predictors (tumor size > 3. 5 cm, AFP > 200 ng/mL and GGT > 53 U/L)

We assigned a prediction score to each independent predictor for MVI according to the β coefficient in multivariate logistic regression model (the β coefficient of each predictor divided by 0.859) (Table 6). The total prediction score of each HCC patient was the sum of the score of each independent predictor, which ranged from 0 to 3 points. Based on ROC curve analysis of the prediction score, the area under receiver operating characteristic (AUROC) was 0.723 (95% CI, 0.655-0.790) in the entire cohort. The optimal cut-off score was 2 on the basis of maximum Youden index value. The sensitivity, specificity, PPV and NPV were 69.0% (95% CI, 58.1-78.5%), 70.6% (95% CI, 62.4-77.8%), 58.3% (95% CI, 48.1-67.9%) and 79.2% (95% CI, 71.2-85.8%) (Figure 3).

**Table 6:** Predictors for microvascular invasion of hepatocellular carcinoma on multivariate logistic regression analysis

Variable	β	OR (95%CI)	*P*	Score
GGT > 53U/L	0.859	2.360 (1.287-4.325)	0.005	1
AFP > 200 ng/mL	0.934	2.544 (1.399-4.628)	0.002	1
Tumor size > 3.5 cm	1.078	2.938 (1.585-5.447)	0.001	1

OR, odds ratio; CI, confidence interval; β, partial regression coefficient.

## DISSCUSION

Early-stage HCC patients are the main candidates for curative treatments and supposed to have relatively good long-term outcomes [11, 12]. However, even with the progress in surgery techniques, the overall survial and recurrence rates are unsatisfactory. Nathan et al reported the median survival time and 5-year survival rate of early-stage HCC patients were only 45 months and 39% [11]. Cai et al investigated that the 5-year overall survival and recurrence-free survival rates were 77.7% and 59.9% in small HCC patients (maximum tumor size ≤ 3 cm) after resection [3]. Tumor size, vascular invasion, histological grade and treatment strategy have been considered to be independent risk factors influencing the prognosis of early-stage HCC [12–14]. In the present research, we identified a total of 233 early-stage HCC patients (solitary tumor, tumor size ≤ 5 cm and no macrovascular invasion). The 5-year overall survival and recurrence-free survival rates were 63.5% and 39.2%, respectively. Cox multivariate regression analysis showed that ICG-R15, BCLC staging and MVI were independent risk factors for the overall survival rate. While type of resection and MVI were independent risk factors for the recurrence-free survival rate.

A systematic review including 20 observational studies revealed MVI was closely related to poor prognosis of HCC patients [15]. However, the presence of MVI was reported to be associated with tumor size [9, 16]. Ahn et al found that patients with tumor size > 5 cm had higher risk of MVI than those with tumor size ≤ 5 cm [17]. In the previous study of our group, the prevalence of MVI was 55.7% in solitary HCC patients with tumor size > 5 cm [18]. In patients with advanced HCC, it is generally accepted that MVI is the main cause for metastasis and recurrence after hepatectomy [10, 19]. But there is controversial about the significance of MVI on the prognosis of early-stage HCC. Shindoh et al suggested small HCC (≤ 2 cm) was related to a good prognosis without regard to the presence of MVI [20]. Nevertheless, Du et al claimed MVI is a poorer prognostic predictor for small HCC (tumor size ≤ 3 cm) [21]. In our research, the prevalence of MVI was 37.3% in early-stage HCC. The 1-, 3-, 5-year overall survival and recurrence-free survival rates of patients with MVI were significantly poorer than those of patients without MVI. MVI was the only independent risk factor for both the overall survival and recurrence-free survival rates in this study.

We investigated the recurrence patterns of HCC patients with and without MVI in the present research. Though there were no significant differences in the intrahepatic, extrahepatic and numbers of recurrence, the median time to recurrence in patients with MVI after hepatectomy was significantly shorter than that in patients without MVI. Compared with patients without MVI, the marginal recurrence in patients with MVI was higher. These results suggested that HCC patients with MVI were prone to early recurrence and marginal recurrence after hepatectomy. MVI was defined as the invasion of tumor cells in a portal vein, hepatic vein, or a large capsular vessel of the surrounding hepatic tissue [15]. The presence of MVI has been considered to be the first-step of hematogenous metastasis in HCC, which is related to aggressiveness of HCC. Even in the early stage of HCC patients, the invasive tumor cells could spread through invading the intrahepatic vascular system, especially the portal vein and its branches. Futhermore, Shi et al reported MVI usually were found in the normal liver tissue within 2 cm away from the tumor edge [22]. If the extent of liver resection is not adequate, the residual MVI near the surgical margin may be the important cause for intrahepatic early recurrence and marginal recurrence in HCC patients.

The important pathological characteristic of MVI is that tumor cells spread through portal venous and hepatic venous branches. Therefore anatomical resection based on Couinaud’s segment is recommended for HCC patients with MVI. Compared with non-anatomical resection, anatomical resection could remove the portal tributaries bearing the tumor completely, which is more effective to eradicate intrahepatic MVI to reduce the recurrence rate. In our series, anatomical resection significantly improved the overall survival and recurrence-free survival rates in the early-stage HCC patients with MVI. Similar results were not found in patients without MVI. In a multi-center retrospective study from Italy and China, Cucchetti et al investigated that anatomical resection significantly reduced the recurernce rate in 153 early HCC patients with MVI [23]. Shindoh et al aslo revealed the prognostic superiority of anatomical resection was confirmed only in patients with histopathological evidence of MVI [24]. These results supported our conclusion. Thus, if patients has an adequate future liver remnant, anatomical resection should be first taken into consideration in HCC patients with MVI.

Unfortunately, MVI is only confirmed after hepatectomy by histopathological diagnosis, which limits its widespread use in the choice of surgical procedure. Therefore, it is valuable to identify the predictors for MVI preoperatively. By retrospectively investigating preoperative characteristics of patients, We identified three independent predictors for MVI: tumor size > 3.5cm, AFP > 200 ng/ml and GGT > 53U/L. Tumor size is considered as the most valuable predictor for MVI in HCC patients [10, 25]. The risk of MVI continues to rise with the increase of tumor size. Our research showed tumor size was still a strong predictor for MVI, even in solitary HCC patients with tumor size ≤ 5 cm. The significant cut-off value of tumor size was identifed as 3.5 cm. The elevated level of AFP have been reported to be associated with MVI [26, 27]. Schlichtemeier et al investigated 125 HCC patients who underwent liver resection, and showed that a serum AFP level ≥ 400 ng/ml was independently associated with MVI [28]. The cut-off value of AFP was higher than ours because only early-stage HCC patients were included in our research. Circulating tumor cells is considered as one possible mechanism of MVI [29, 30]. Jin et al reported that the high AFP mRNA level of circulating tumor cells was a valuable predictor for vascular invasion of HCC after hepatectomy [31]. The results might explain the relation between high level of AFP and MVI. Aberrant expression of GGT has been found in HCC and plays an important role in tumor formation and metastasis [32]. A previous research by Ju et al demonstrated the high level of GGT was related to vascular invasion, advanced tumor and tumor size [33]. Zhao et al suggested serum GGT > 130 U/L was an independent predictive factor for MVI in multinodular HCC [10]. While in the present research, our results showed GGT > 53U/L was strongly associated with MVI for early-stage HCC. Based on these three predictors, we built up a prediction scoring system for the risk of MVI. According to the ROC curve, the prevalence of MVI in HCC patients with predictive score ≥ 2 was 58.3%, which was obviously lower than those with predictive score < 2 (20.8%).

The present research has some limitations. First, it is a single-center and retrospective research. Therefore, it was subject to potential bias that might preclude definite conclusions to be drawn. The prognostic value and preoperative predictors of MVI require prospective and multicenter validations. Second, thin-slice contrast-enhanced CT and gadolinium-ethoxybenzyl-diethylenetriamine pentaacetic acid (Gd-EOB-DTPA) MRI have been reported to be helpful for predicting MVI preoperatively [29, 34]. Because these imaging techniques were widely applied in recent years, detailed imaging imformation in early patients of our cohort were not included in the present research.

In conclusion, our study indicated that MVI was an independent risk factor for the overall survival and recurrence-free survival rates of solitary HCC patients with tumor size ≤ 5 cm after hepatectomy. For HCC patients with MVI, anatomical resection could significantly improve the overall survival and recurrence-free survival rates. Based on tumor size > 3.5cm, AFP > 200 ng/ml and GGT > 53U/L, the prediction scoring model are valuable for the preoperative prediction of MVI.

## PATIENTS AND METHODS

### Study population

A total of 307 consecutive HCC patients (size ≤ 5 cm) underwent curative hepatectomy in the Department of Hepatobiliary Surgery at Nanjing Drum Tower Hospital between January 2004 and December 2013. Patients met the following criteria were enrolled in the present study: (1) no evidence of macroscopic vascular invasion, (2) solitary tumor, (3) Child-Pugh A/B, (4) R0 tumor resection, (5) no any preoperative anticancer treatments, (6) no history of other cancers, (7) complete clinical and pathological data. Eventually, a total of 233 early-stage HCC patients were enrolled. The present study was carried out in accordance with the Declaration of Helsinki revised in 1983. The retrospective study was approved and exempted from the requirement to obtain informed consent by the Committee on Medical Ethics of Nanjing Drum Tower Hospital.

### Clinical and pathological characteristics

Preoperative clinical data and operation information were retrospectively reviewed from our HCC database, including age, gender, serum hepatitis B surface antigen (HBsAg), Child-Pugh grade, ICG-R15, BCLC staging, serum alanine aminotransferase (ALT), serum aspartate aminotransferase (AST), gamma glutamyl transpeptidase (GGT), alkaline phosphatase (AKP), serum total bilirubin (TB), direct bilirubin (DB), serum albumin (ALB), alpha-fetoprotein (AFP), platelet count (PLT), international normalized ratio (INR), type of operation, operation time, blood loss and blood transfusions (Table 1). Anatomical resection was charactered as any type of complete excision at least one segment based on Couinaud’s classification, including segmentectomy, sectoriectomy and hemihepatectomy. Non-anatomical resection was defined as local resection or enucleation without regard to the Couinaud’s segmental and sectoral structure. MVI was evaluated based on all the liver slices of resected specimens. MVI was defined as the invasion of tumor cells in a portal vein, hepatic vein, or a large capsular vessel of the surrounding hepatic tissue, partially or totally lined by endothelial cells that were visible only on microscopy [19]. The extent of tumor differentiation was evaluated as well, moderate and poor according to Edmondson-Steiner grading system [35].

### Patient follow-up

Patients were followed up systematically by the levels of AFP, liver function and abdominal ultrasonography every 2 month after discharge. Contrast-enhanced computed tomography (CT) or enhanced magnetic resonance imaging (MRI) was performed every 4 months. Recurrence should be confirmed by at least two imaging modalities, such as CT and MRI. The time to recurrence, site of recurrence and number of recurrence were recorded. The site of recurrence was divided into intrahepatic and extrahepatic recurrence. Intrahepatic recurrence was divided into recurrence at surgical margin, ipsilateral hemiliver and contralateral hemiliver. The number of recurrence was divided into solitary and multiple. If the recurrence was detected, further treatment such as second hepatectomy, local ablation, transcatheter arterial chemoembolization (TACE), or other therapeutic modalities, including molecular targeted therapy would be undertaken. Overall survival was defined as the time interval between the operation and the date of the death. Recurrence-free survival was defined as the period after the operation when a recurrence could be detected. Follow-up data were collected until December 31, 2015.

### Statistical analysis

Categorical data were compared by the chi-square test or Fisher’s exact test. The survival analyses were performed according to the Kaplan-Meier survival curves and compared by the log-rank test. Prognostic risk factors were analyzed by univariate and multivariate Cox proportional hazards models. The predictors for MVI were identified by univariate logistic regression analysis. Subsequently, the significant predictors (*P* < 0.10) were evaluated by multivariate logistic regression analysis to identify the valuable independent predictors for MVI. The assigning score of each predictor was determined according to the β coefficient in multivariate logistic regression model [36]. The cut-off value of predictive score was calculated by receiver operating characteristic (ROC) curve. The sensitivity, specificity, positive predictive value (PPV) and negative predictive value (NPV) were calculated. For all tests, *P* < 0.05 were considered statistically significant. Statistical analysis was performed using SPSS version 21.0 (SPSS Inc., Chicago, IL).
